# Structural and UV-blocking properties of carboxymethyl cellulose sodium/CuO nanocomposite films

**DOI:** 10.1038/s41598-023-28032-1

**Published:** 2023-01-20

**Authors:** Rania Badry, Mahmoud M. El-Nahass, Nadra Nada, Hanan Elhaes, Medhat A. Ibrahim

**Affiliations:** 1 Physics Department, Faculty of Women for Arts, Science and Education, Ain Shams University, Cairo, 11757 Egypt; 2 Physics Department, Faculty of Education, Ain Shams University, Roxy, Cairo, Egypt; 3 Molecular Spectroscopy and Modeling Unit, Spectroscopy Department, National Research Centre, 33 El-Bohouth St., Dokki, Giza, 12622 Egypt

**Keywords:** Materials science, Physics

## Abstract

Nanoparticles have made a substantial contribution to the field of skincare products with UV filters in preserving human skin from sun damage. The current study aims to create new polymer nanocomposite filters for the efficient block of UV light that results from the stratospheric ozone layer loss. The casting approach was used to add various mass fractions of copper oxide nanoparticles (CuO-NPs) to a solution of carboxymethyl cellulose (CMC). The amorphous nature of CMC was revealed by XRD analysis, with the intensity of the typical peak of virgin polymer in the nanocomposite spectrum decreasing dramatically as the doping amount was increased. The FTIR spectra revealed the functional groups of CMC and the good interaction between the CMC chain and CuO-NPs. Optical experiments revealed that the optical transmittance of pure CMC was over 80%, whereas it dropped to 1% when CuO-NPs content was increased to 8 wt.%. Surprisingly, the inclusion of CuO-NPs considerably improved the UV blocking property of the films extended from the UV region (both UV-A: 320–400 nm and UV-B: 280–320 nm) to the visible region. Optical band gap of CMC decreased sharply with increasing CuO concentration. The tunable optical characteristics can be utilized in UV- blocking filters and various optoelectronics applications.

## Introduction

Sunlight has a variety of positive effects in humans, including the production of vitamin D and the reduction of β-endorphin expression, both of which improve well-being^1^. The loss of the stratospheric ozone layer has resulted in a gradual increase in UV radiation in recent decades. This increases the number of negative health effects on humans, such as skin burns, photoaging, immune system damage, and skin cancer^1,2^. As a result, the number of skincare products with UV filters has risen rapidly, helping to protect human skin from sun damage. There were 26.9 million tonnes of products used worldwide that had UV filters, with modern consumption presumably being substantially greater^3^. UV filters are often used in a variety of personal care items, including lotions, shampoos, creams, aftershaves, and cosmetics^4–6^.

Photo protective techniques predate the introduction of the first sunscreen in the 1900s, and ancient civilizations have long employed plant extracts to protect their skin from sunburns^7^. Many of the photo protective chemical components found in natural extracts are now used in sunscreens^7^. In fact, the majority of UV filters are derived from natural sources, such as plant, animal, or mineral compounds^7^. The photo protective effect of benzyl salicylate against UV-B radiation was discovered in 1928, but it was first commercialized in 1935 in the first sunscreen, "Ambre Solaire"^8,9^.

Based on their capacity to absorb UV radiation (UVR), UV filters are classified as UV-A, UV-B, or broad-spectrum UV filters (UV-A and UV-B)^10^. UV-A wavelengths ranged from 320 to 400 nm. Additionally, these groups can be divided into organic and inorganic filters, with organic filters having the ability to just absorb UVR and inorganic filters having the ability to reflect and scatter UVR^11^.

The safety of UV filters for both people and the environment has come under scrutiny recently. Further investigation into the effectiveness of UV filters used topically on the skin has revealed a progressive decline in their UV-protective ability that cannot just be attributable to photo-induced degradation. Researchers that looked into the causes of this behavior came to the conclusion that UV filters might be routinely absorbed via the skin's surface or even discharged during bathing and washing activities^12,13^. Numerous studies were conducted in an effort to determine the extent and impact of skin penetration as well as accumulation in the aquatic environment as a result of these discoveries^14–16^. The initial demand for product certification has been expanded to include trustworthy analytical methods to identify these compounds at low concentration levels and in environmental samples due to the necessity for a more thorough inquiry into the behavior of UV filters^7,17^. As a result, over the past 20 years, researchers have studied new natural substances^18,19^, synthetic derivatives^20,21^, as well as the application of nanotechnology approaches^7^, as means of identifying more effective, new, safe, and stable UV filters.

In 2022, Nasrallah, D. A., and Ibrahim M. A. studied the effect of silver doped hydroxyapatite NPs on the structural, optical, and dielectric characteristics of a PVA/ CMC polymer blend. They found that the optical transmittance of the pure PVA/ CMC blend was nearly 90%, while it decreased to 50% with increasing AgHA-NPs contents up to 40 wt.%^22^. In another study, Bian et al. (2021) stated that increasing lignin concentration in the lignocellulose nanofiber (LCNF) film improves its UV-blocking capacity and thermal stability^23^.

In comparison to organic UV filters, inorganic nanoparticles have generally been considered to be more stable and secure anti-UV compounds^24^. Polymer matrix nanocomposites, which display distinct physicochemical properties by incorporating inorganic fillers into polymeric chains, have gotten a lot of attention because of their various industrial applications in drug delivery, water treatment, food processing, and aeronautical and aerospace structures^25–27^. This nanocomposite combines polymer advantages with semiconductor nanoparticles' size-tunable optical, electrical, catalytic, and other capabilities.

Carboxymethyl cellulose sodium (CMC), one of the most important cellulose derivatives, is typically made by reacting alkali cellulose with monochloroacetate or its sodium salt in an organic medium. CMC has a number of desirable characteristics, including foaming, emulsification, suspension, and water retention. As a result, it's used in a variety of industries, including food technology, as a viscosity modifier, thickener, and stabilizer^28–30^. It is also found in a variety of nonfood goods, such as toothpaste, detergents, water-based paints, textile sizing, electrical components, and a variety of paper products. Stronger inter- and intra-hydrogen bonding can be formed due to the presence of the hydroxyl group (OH) within the chain backbone of the CMC structure^31–33^.

The purpose of this research is to improve the optical characteristics of a CMC by adding CuO-NPs to it. To the best of our knowledge, no research has ever been done on how the concentration of CuO-NPs affects the optical characteristics and capacity of UV blocking of CMC. CuO-NPs is chosen because it has numerous unique physical and chemical characteristics. These qualities include its low cost, availability, nontoxicity, and chemical stability^34–36^. Additionally, due to its intermediate energy band gap (1.2–2.6 eV), it is a strong contender for use in a variety of applications, including data storage, sensors, photocatalysis, optoelectronic devices, and others^37–39^. Due to its ease of use and low cost, the solution casting process was used to manufacture various weights of CuO-NPs packed CMC in order to take advantage of their structural and optical features. X-ray diffraction (XRD), Fourier transform infrared (FTIR) spectroscopy, and ultraviolet–visible-near-IR (UV–Vis-NIR) spectroscopy measurements have been used to examine the effects of the weight ratio percentages (wt%) of the nanofiller on the CMC’s structural and optical characteristics, respectively. The results show that CuO-NPs filling can be used to adjust the optical parameters of CMC to make it a significant candidate for blocking the UV radiation.

## Materials and methods

### Materials

Carboxymethyl cellulose sodium (CMC) with Mw = 2.5 × 10^5^ g/mol was purchased from K. Patel Chemo pharma PVT, (India), while copper chloride was purchased from Sigma-Aldrich. El Nasr Pharmaceutical Chemicals Co., Cairo, Egypt, provided sodium hydroxide and ethanol.

### Preparation of CuO-NPs

CuO-NPs were synthesised by mixing 3.4 g of copper chloride in 100 ml of distilled water (DW) using a magnetic stirrer at 50 °C until a homogenous solution was obtained. Under continuous stirring, aqueous sodium hydroxide solution (0.4 M NaOH in 100 ml DW) was added drop by drop to the copper chloride solution until a black precipitate formed and stirred at 80 °C for 4 h. The black precipitate was washed many times with DW and ethanol, dried in an oven at 80 °C for 2 h, and then calcinated for 2 h at 500 °C to obtain copper oxide nanoparticles.

### Preparation of CMC/CuO nanocomposite samples

The solution casting approach was used to synthesis pure CMC and its nanocomposite films. To begin, 0.5 g of carboxylmethyl cellulose was dissolved in 100 ml of distilled water with constant stirring at 40 °C for 4 h to produce a homogeneous CMC solution. After cooling the solution to room temperature and removing any air bubbles, the film solution was cast into two glass petri dishes. The solution was dried in an air-circulating oven at 40 °C for 24 h. The dried films were removed and stored in the sealed bag until the test. This command was used to code the film as CMC. The optical background was measured and subtracted from the sample’s spectrum.

By adding different proportions of CuO-NPs (2, 4, 6, and 8 wt.%) to the previously prepared CMC solution, as tabulated in Table 1, CMC/CuO nanocomposite films were created. The mixture was treated by sonication at 50 °C and turned dark grey after another 15 min, so it was placed into glass petri dishes and dried in a 40 °C oven for 24 h. Finally, the films were carefully removed from the petri dishes. The thickness of the films was measured with a digital micrometer with a precision of 0.001 mm, and the mean values of three random points for each sample were calculated as presented in Table 1.

**Table 1:** Sample coding according to CuO-NPs concentration and the thickness of CMC and CMC/CuO nanocomposite films.

Structure	CMC (g)	CuO (g)	D.W. (ml)	Thickness (mm)
CMC	0.250	0.000	50	0.032
B1	0.245	0.005		0.030
B2	0.240	0.010		0.027
B3	0.235	0.015		0.028
B4	0.230	0.020		0.038

### Characterization techniques

To analyze the crystalline structures of the generated nanocomposite samples of CMC and CuO, XRD spectra in the range 5–90° were obtained using a Philips X-ray diffractometer with a step size of 0.026° and a Cu-K_α1–2_ source of λ = 1.54 A°, 45 kV, and 40 mA.

Hand grinding with a mortar and pestle provides powdered XRD samples (CuO-NPs). To minimize sample loss during grinding and to mitigate structural damage to the phases in the sample caused by grinding, samples were typically crushed in a liquid medium such as ethanol. Using Fourier transform infrared spectroscopy (Pruker vertex 70) in the wavenumber range (4000–400 cm^−1^), the molecular structure of CMC and CuO-NPs was investigated. The penetration depth of the diamond ATR accessory with a type II alpha diamond crystal is 2 µm. The spectra were collected at a spectral resolution of 4 cm^−1^. Each spectrum received a total of 35 scans. The same settings were used to test the background versus air with a resolution of 4 cm^−1^. Samples were used as it is without any preparation. CuO-NPs were investigated using high resolution transmission electron microscopy (JEOL, JEM-2010-F), operated at an accelerating voltage of 200 kV, to learn more about the size and morphology of the produced CuO nanoparticles. Using a vortex mixer, the particles were dissolved in ethanol before being put onto a gold grid (300 mesh, TED PELLA, INC) for observation. The optical properties were investigated in the wavelength range 200–1000 nm using an ultraviolet/visible spectrophotometer (UV/Vis., V-570 UV/VIS/NIR, JASCO, Japan) at room temperature. Distilled water was used to provide a suspended CuO- NPs for optical analysis.

### Ethical approval

This work is not applicable for both human and/ or animal studies.

## Results and discussion

### X-ray diffraction analysis (XRD)

**Figure 1:**
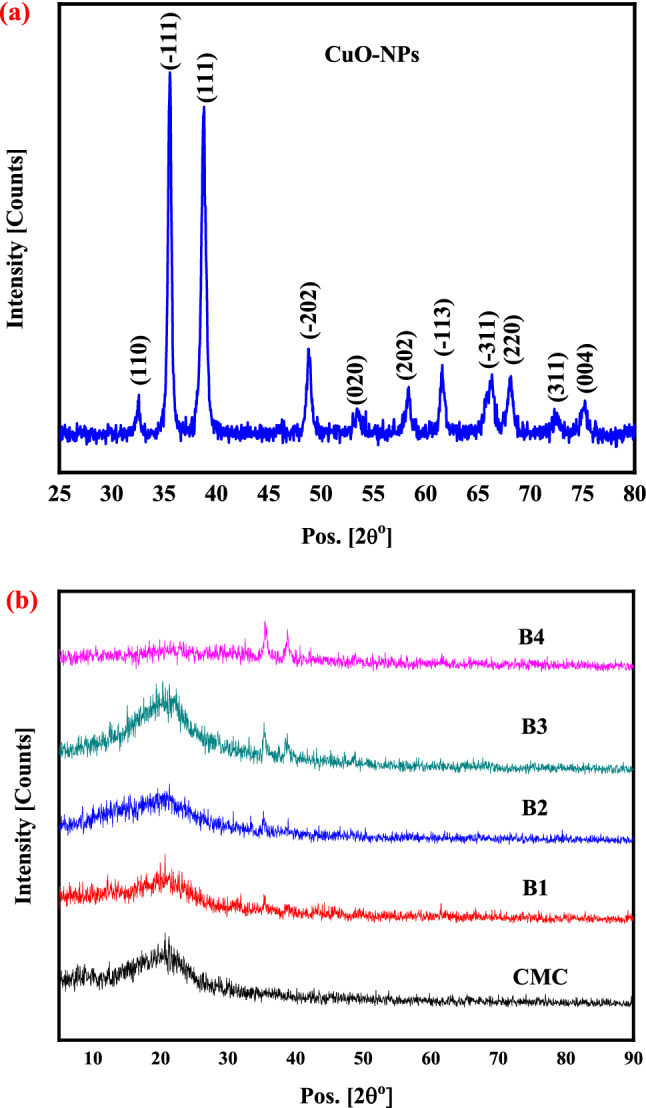
X-ray diffraction pattern of: (**a**) CuO-NPs and (**b**) CMC nanocomposite films including various CuO-NPs concentrations (0, 2, 4, 6 and 8 wt. %).

X-ray diffraction is the most commonly used technique for evaluating the structure and purity of the samples. The XRD pattern of CuO-NPs was collected in the range of 2θ = 25°- 85° as presented in Fig. 1a, with peaks at 2θ = 32.51°, 35.52°, 38.76°, 48.82°, 53.46°, 58.31°, 61.56°, 66.11°, 68.04°, 72.44° and 75.09° corresponding to the diffraction planes: (110), (−111), (111), (−202), (020), (202), (−113), (−311), (220), (311), and (004), respectively. The sharp peaks observed confirm the good crystallinity of the prepared CuO-NPs. The obtained XRD pattern of CuO-NPs is well indexed to the monoclinic structure of CuO-NPs (tenorite) in the P63/m space group (ICDD card No. 01–089-5895) with superior crystal quality. The absence of any secondary phase, such as Cu(OH)_2_ or Cu_2_O, indicates the presence of only CuO-NPs and the high quality of the nanoparticles synthesized. The Bragg law was used to compute the distance between the crystallographic planes (d_hkl_):

1 $$n\lambda \, = \, 2d \, \sin \theta$$

where, n is the order of X-rays diffraction, λ is the wave's incidence wavelength, d is the plane separation between lattices, and θ is the scattering angle. The diffraction peak’s broadening can be utilized to estimate the crystallite size of powder samples. In addition, the crystallite size was estimated utilizing the Debye–Scherrer equation^39^:

2 $$D=\frac{K\lambda }{\beta Cos\theta }$$

where, D is the crystalline size in nanometers, k is the Scherrer's constant (0.9), λ is the X-Ray wavelength, θ is peak position, and β is the full width at half-maximum (FWHM) of the diffraction peak. The average crystallite size of the prepared CuO-NPs was calculated and equals 34.01 nm.

The following equations were used to calculate micro strain (ε), dislocation density (δ), and the number of crystallites (N_c_) based on the obtained data (see Table 2).

**Table 2:** Crystallite size (D), dislocation density (δ), micro strain (ε), and number of crystallites (N_c_) for the most prominent peak (−111) of CuO-NPs and CMC/CuO nanocomposite films.

Structure	D (nm)	δ(nm^-2^) × 10^–3^	ϵ × 10^–3^	Nc
CuO	33.60	2.09	5.20	957.24
B1	104.23	0.09	1.09	0.02
B2	35.84	0.78	3.19	0.59
B3	28.08	1.27	4.07	1.26
B4	14.93	4.49	7.61	11.43

3 $$\varepsilon =\frac{\beta cos\theta }{4}$$

4 $$\delta =\frac{1}{{D}^{2}}$$

5 $$\mathrm{Nc}=\frac{t}{{D}^{3}}$$

The XRD patterns of a virgin CMC and CMC with different quantities of CuO-NPs are shown in Fig. 1b. It can be seen that the CMC matrix and loading CuO-NPs films have a broad peak near 2θ = 20° due to the amorphous nature of the CMC. It could also be deduced that the intensity of this peak decreases with the addition of CuO-NPs. The changes could be attributed to the interaction between the CMC and the CuO-NPs. Furthermore, more inter- and intramolecular attraction could occur, softening the polymer backbone. It is well known that peak broadening (observed in Fig. 1b) occurs as a result of two major effects: crystallite size reduction and microstrain generation in the material’s crystal structure. As a result, by analyzing peak broadening, it is possible to gain insight into the material's associated properties. Similar results were reported for CMC films^40^.

The addition of CuO-NPs as nanofiller to a pristine CMC matrix was confirmed by the presence of a number of remarkable peaks of CuO-NPs, an increase in peak intensity with increasing CuO content, and a decrease in CMC diffraction peak intensity (see Fig. 1b). Furthermore, substantial reflections can be seen in the XRD measurements along the (-111), (111) and (-113) planes of all nanocomposites. This reflection planes corresponds to the sharp diffraction peaks around 35.53°, 38.79°, and 61.62°. Moreover, as the CuO-NPs concentration in the polymer matrix increased to 8 wt.%, the XRD intensity along these reflections increased. Additionally, micro strain, dislocation density, and number of crystallites were calculated for the most prominent peak in the nanocomposite films (see Table 2). The values of dislocation density and micro strain increased with increasing CuO-NPs concentration, as shown in the table. Also, the sample abbreviated as B4 (contains 8wt.% CuO) possesses a dislocation density and micro strain higher than that of CuO-NPs. Furthermore, as demonstrated by XRD intensity fluctuations in doped films, variations in vacancy and defect density cause significant changes in grain boundaries and thus lattice strain.

The number of crystallites in the nanocomposite films increased as the CuO-NPs content increased. The number of crystallites ranged from 0.02 to 11.43. As the CuO-NPs concentration increased, the broadening of the diffraction peak (-111) narrowed, as shown in Fig. 1b, as the number of crystallites (Nc) increased. These results confirm the influence of CuO-NPs on the crystallinity of pure CMC. The reason for increasing the number of crystallites with CuO-NPs addition is the decrease in crystallite size, where the number of crystallites is inversely proportional to the crystallite size as presented in Eq. (5). This reflects the good dispersion of CuO-NPs in the nanocomposite.

### Fourier transform infrared spectroscopy (FTIR) analysis

**Figure 2:**
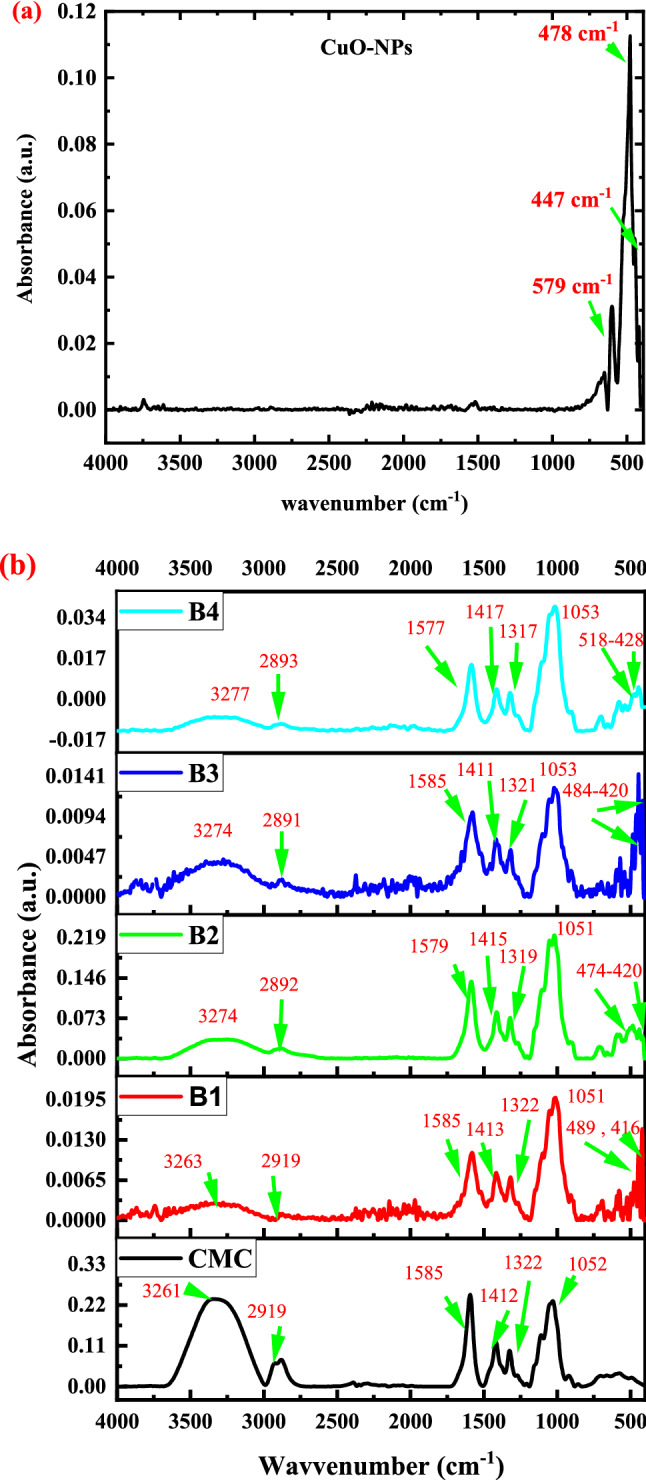
FTIR spectra of (**a**) CuO nanoparticles and (**b**) pure CMC and CMC filled with different concentrations of CuO-NPs (2, 4, 6 and 8 wt.%).

Understanding the chemical and structural properties of the synthesized CMC, as well as the impact of CuO-NPs, is critical and is satisfied utilizing FTIR analysis. The CuO-NPs can be identified by the tree distinctive bands found at 447 cm^−1^, 478 cm^-1^, and 579 cm^−1^ (see Fig. 2a). The high-frequency mode at 579 cm^−1^ and the peak at 478 cm^−1^ can be attributed to the Cu–O stretching vibration. Additionally, no other IR active mode was seen between 600 and 660 cm^−1^, ruling out the possibility of another phase, such as Cu_2_O^41^.

The FTIR spectra of a pure CMC and a CMC containing various concentrations of CuO-NPs (2, 4, 6 and 8 wt.%) are shown in Fig. 2b. The key feature bands of the virgin CMC are revealed in the FTIR spectrum. The broad band at 3261 cm^−1^ may be assigned to the O–H stretching vibration. The band at 2919 cm^−1^ may be due to the C–H asymmetrical stretching vibrations of the CH_2_. Carboxyl group (COO) asymmetric stretching and CH_2_ scissoring vibration are responsible for the observed bands at 1585 and 1412 cm^−1^, respectively. The presence of OH bending motion is demonstrated by the band at 1322 cm^−1^.

Furthermore, the band at 1014 cm^−1^ was attributed to the C–O stretching, whereas the band at 906 cm^−1^ was caused by CH_2_ rocking. The creation of intermolecular hydrogen bonding between the CMC and CuO-NPs of the nanocomposite films was expected, according to Saadiah et al.^42^, and so these actually occur at the bending OH region and asymmetric COO functional group of CMC. The integration of CuO-NPs influences the functional groups of CMC as a result of the interactions between these groups and CuO-NPs, as presented in Fig. 2b. With the addition of CuO-NPs, the strength of the OH stretching vibration band of the pure film at 3261 cm^−1^ reduced and shifted to a higher wave number, as seen in Fig. 2b. Additionally, the figure shows that all samples possess high intensity bands assigned to CuO-NPs from 400 to 600 cm^−1^ which may be due to the structural changes of CMC with CuO addition and the increased concentration of CuO-NPs. All of this evidence supports the formation of a hydrogen bond between CuO-NPs and pristine CMC, as well as providing important structural modification information.

With increasing CuO-NPs content, the intensity of C–H stretching, COO stretching, and CH–O–CH_2_ stretching increased and shifted toward a higher wave number (see Table 3). Additionally, the intensity of CMC’s absorption band assigned to the C–O–C bending vibration (1053 cm^−1^) was also increased, and this confirms that strong interaction occurred between CMC and CuO via this band. This reflects the good interaction between CMC chains and CuO-NPs supports the results of XRD analysis. Moreover, the strong absorption bands at 419 cm^−1^ revealed the stretching mode of Cu–O bond vibration in the monoclinic phase of CuO-NPs. All of this evidence supports the formation of a hydrogen connection between CuO-NPs and virgin CMC, as well as providing structural information^42^.

**Table 3:** FTIR band assignments for pure CMC and CMC filled with different concentrations of CuO-NPs.

Wavenumber (cm^-1^)					Band assignment
CMC	B1	B2	B3	B4	
3261	3263	3274	3274	3277	Stretching of OH group
2919	2919	2892	2891	2893	CH stretching of the CH_2_ groups
1585	1585	1579	1585	1577	Carboxylate group (COO^−^) asymmetric stretching
1412	1413	1415	1411	1417	CH_2_ scissoring
1322	1322	1319	1321	1317	OH bending
1052	1051	1051	1053	1053	The C–O–C bending vibration
1019	1020	1012	1020	1020	C–O bond of the CH_2_ OH group
912	911	906	905	904	C–O stretching + CH_2_ rocking motion
657–534	590–518	594–579	669–572	691–584	Ring stretching and deformation
–	489	474	484	518	O–Cu–O
–	416	445	440	446	O–Cu–O
		420	420	428	O–Cu–O

### HRTEM analysis

**Figure 3:**
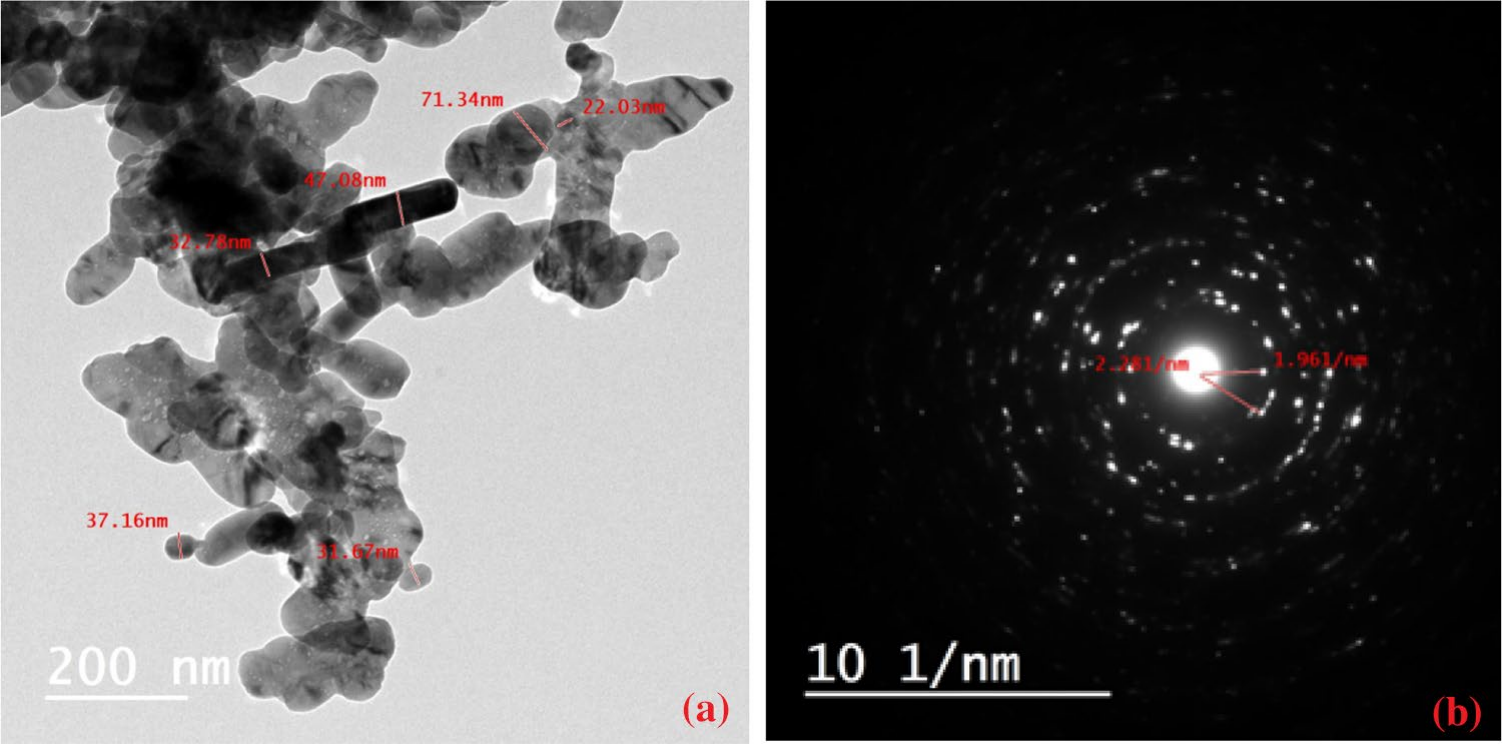
(**a**) TEM images of the agglomerated CuO-NPs and, (**b**) the selected area electron diffraction pattern (SAED).

Figure 3 shows the TEM micrographs of CuO-NPs synthesized by the precipitation method. The deposition of nanoparticle dispersions on the electron-transparent TEM gold grid results in a combination of CuO aggregates and their agglomerates, making it difficult to determine the true CuO-NPs aggregate size (which is obtained by XRD in the present study). The TEM investigation, on the other hand, clearly shows that the agglomerated aggregates are made up of clusters of considerably smaller original crystallites. The average crystallite size of nanoparticles is 40.34 nm as determined by TEM (see Fig. 3a), which is larger than the average crystallite size (34.01 nm) determined by XRD^43^. It is worth noticing that CuO-NPs have a single crystalline pattern with a monoclinic CuO phase, as demonstrated in Fig. 3b. The d-spacing corresponds to the reflection planes of [110] and [−202].

### UV-blocking capacity

**Figure 4:**
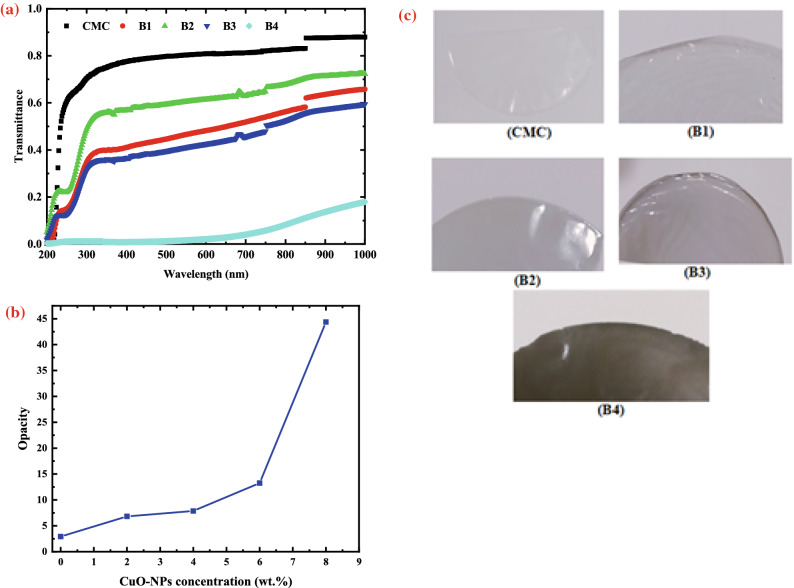
(**a**) UV/Vis-Transmittance spectra for pure CMC and CMC loaded with (2, 4, 6 and 8 wt.% CuO-NPs), (**b**) variation of opacity with CuO concentration and **(c)** visual examination of CMC/CuO nanocomposite films.

A UV–Vis spectrophotometer was used to measure the transmittance of the produced films at wavelengths ranging from 200 to 1000 nm (Fig. 4a). The results revealed that the CMC film containing 8 wt.% of CuO had a high UV-blocking capability, reaching 99% UV-B and UV-A blocking.

Again, we believe that the addition of CuO-NPs to the CMC solution had a significant impact on its ability to absorb UV-light in this investigation, since the addition of CuO-NPs to the CMC solution improved UV-blocking performance, particularly in the UV-A area. Figure 4a shows how 8 wt.% CuO-NPs additions can block 100% of UV-A (320–400 nm) and UV-B (280–320 nm). In comparison to 8 wt.% CuO-NPs, the addition of 2, 4, and 6 wt.% CuO-NPs resulted in a decrease in transparency, even though the UV-C (from 200 to 280 nm) and UV-B regions were not completely blocked at the CuO-NP concentrations used. As a result, CMC/ CuO with a concentration of 8 wt.% CuO-NPs was discovered to be the optimal percentage for successfully blocking UV radiation. These findings were in line with a prior study that found that adding lignin to cellulose-based films increases UV protection while decreasing transparency^21^. Sadeghifar et al. developed a commercial cellulose film containing 2% lignin that blocked 100% of UV-B and more than 90% of UV-A^21^.Similarly, when lignin was added to the PVA film at a higher concentration (3 wt%), effective UV-B blocking was found, but UV-A transmittance was not^43,44^.

At 600 nm, Fig. 4b demonstrates the relationship of opacity for undoped CMC and nanocomposite films. Equation (6) was used to compute the opacity of the films^45^:

6 $$Opacity= \frac{{A}_{600}}{X}$$

where, A_600_ is the absorbance at 600 nm and X is the film thickness (mm). A higher opacity number indicates that a film is less transparent. For all samples, the optical transmittance rose as the wavelength was raised. A simple visual examination of the prepared films reveals that their colour gradually changes from transparent to relatively dark grey and finally to black, with the CMC film being more transparent (lower opacity value) than films incorporating CuO-NPs (Fig. 4c). The percentage of light transmittance at wavelengths of 300 and 600 nm, on the other hand, is reported in Table 4.

**Table 4:** Percentage transmittance T%, at two different wavelengths, for pure CMC film and its filled samples with different concentrations of CuO- NPs.

CMC loaded with CuO-NPs (wt.%)	T% at 300 nm	T% at 600 nm
0	70.397	80.865
2	34.73	48.19
4	48.878	61.359
6	31.199	42.546
8	1.101	2.063

It was discovered that, when CMC is combined with CuO-NPs, the outcome is a decrease in the T% at λ = 300 and 600 nm, as it drops to around 1.10089% and 2.06329%, respectively. The transmittance of the composite is affected by a variety of factors, including intrinsic material properties such as particle size and refractive index of the components, as well as composite fabrication factors such as nanocomposite thickness, filler concentration, surface roughness, and particle dispersion state. Thus, particles with sizes larger than the visible wavelength would obstruct light, leading to translucent or opaque films. Due to the absence of light-blocking particles, the pure CMC film has the highest percent transmittance (Fig. 4a). Another possible explanation for the decreased transparency of the nanocomposite films is that the agglomerations of CuO-NPs became a barrier to the penetration light. When nanofillers are added to a transparent polymer matrix, transparency is lost, resulting in an opaque nanocomposite. Actually, the matrix can be thought as a non-absorbing, uniform medium through which light flows without deflection. Light interferes with the particles when different refractive index fillers are added. Blocking or filtering of UV is caused by two processes: UV light absorption and UV light scattering. The Rayleigh theory describes how particle size and the difference in refractive index between particles and medium affect light scattering^40^.

The outcomes revealed that the addition of CuO-NPs resulted in a significant decrease in light transmission in both UV and visible light regions. It's worth noting that CuO-NPs’ inclusion had a stronger effect in the UV region than in the visible region, meaning that the film’s UV barrier characteristics were improved while its transparency was lowered. Because UV rays have high oxidizing properties and can have a negative impact on human health and food packaging, blocking UV rays is critical in the food human care and packaging industry^46^.

### Optical band gap determination using the absorption spectra fitting model

In this work, the absorption spectrum model (ASF) was applied to calculate optical energy band deviations for the nanocomposites studied, utilizing the UV absorption spectra measured^47^. The ability to calculate the optical gap energy (${E}_{Opt}^{ASF}$) of the samples without having to assess their thickness distinguished this method. The calculation is solely based on the samples' absorbance data. According to Souri and Shomalian, the optical gap energy depends on the wavelength of the incident photon and is expressed as follows:

7 $${E}_{Opt}^{ASF}= \frac{1240}{{\lambda }_{Opt}^{ASF}}$$

**Figure 5:**
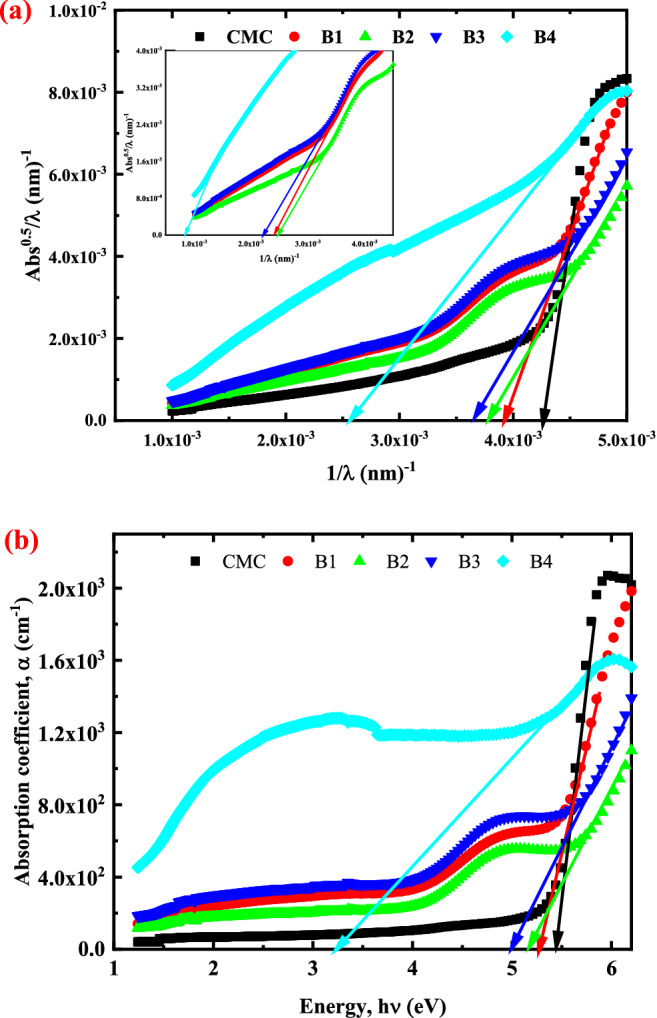
(**a**) Optical band gap determination using the ASF model for pure CMC and CMC loaded with different weight percentages of CuO-NPs and (**b**) Fluctuation in the absorption coefficient ( α) as a function of photon energy (hν) for CMC and CMC filled with 2, 4, 6 and 8 wt.% of CuO-NPs.

Figure 5a shows how, for indirect transitions, (Abs^1/2^)/λ vary with (λ)^-1^. The calculated and listed values of ${E}_{Opt}^{ASF}$ are shown in Table 5. These values are fairly close to those found using Tauc's model^46^. The ${E}_{Opt}^{ASF}$ for indirect transitions of the samples altered with the alteration of CuO-NPs concentration, according to the results, the CuO-NPs inside the bands that create this fluctuation in optical band gap after adding CuO to the CMC matrix. Photons that are incident will be absorbed as a result of these CuO-NPs.

**Table 5:** Indirect allowed band gap values of CMC and CMC doped with 2, 4, 6 and 8 wt. % CuO.

CMC loaded with different wt.% of CuO-NPs	${E}_{g1}^{in}$(eV)	${E}_{g2}^{in}$(eV)	${E}_{Opt 1}^{ASF}$	${E}_{Opt 2}^{ASF}$
0	5.27	–	5.29	–
2	4.87	2.97	4.86	2.99
4	4.66	2.95	4.68	3.06
6	4.49	2.78	4.52	2.75
8	3.14	1.13	3.17	1.04

### The fundamental absorption edge and optical gap

The band gap value of amorphous materials such as polymers must be determined before they can be used. The most common method for inducing the band gap of amorphous materials is to employ optical absorption coefficient measurements to determine the fundamental absorption edge associated with the gap. For accurate determination of the absorption edge, the absorption coefficient was determined using the measured transmittance (T) and reflectance (R) values according to the following equation:

8 $$\mathrm{\alpha }=\frac{1}{\mathrm{d}}\mathrm{ln}[({\left(1-\mathrm{R}\right)}^{2}/2\mathrm{T})+\sqrt{({\left(1-\mathrm{R}\right)}^{4}/4{\mathrm{T}}^{2})+{\mathrm{R}}^{2}]}$$

where d is the thickness of the film. Figure 5b depicts the variation in the absorption coefficient with incident light for pure and doped CMC films. As can be seen, the pure CMC film absorbs significantly in the UV spectral region, showing a high degree of amorphousness. Plotting the coefficient of absorption for all produced films versus the photon energy (hv) revealed the position of the absorption edge, as seen in Fig. 5b. The extrapolation of the linear part of the curve, for all samples, to the energy axis gives the value of the fundamental edge. A continuous red shift may be seen in the graph by increasing the CuO-NPs concentrations in the CMC matrix.

Changes in the electron–hole in the conduction and valence bands are used to explain the absorption edge shift in the UV region. The decrease in the optical bandgap for nanocomposite films anticipates this result. Controlling material use in optical and electrical technologies entails identifying the optical energy gap, as previously discussed. Indeed, Mott and Davis^48^ claim that the optical gap E_g_ for non-crystalline materials may be calculated using the following formula:

9 $$\left( {\alpha {\text{h}}\nu } \right) \, = {\text{ B}}\left( {{\text{h}}\nu - {\text{E}}_{{\text{g}}} } \right)^{{\text{r}}}$$

where α is the absorption coefficient, h is the plank’s constant, ν is the incident frequency, B is a constant, E_g_ is the optical band gap, and r is a constant that depends on the transition type; r = 1/2 for allowed direct transition and 3/2 for forbidden direct transition, and r = 2 and 3 for indirect allowed and forbidden transitions, respectively.

All of these parameters reduce significantly when CuO-NPs concentration increased. This reduction occurs when CuO-NPs with a low energy band gap are added to the high bandgap energy CMC matrix.

**Figure 6:**
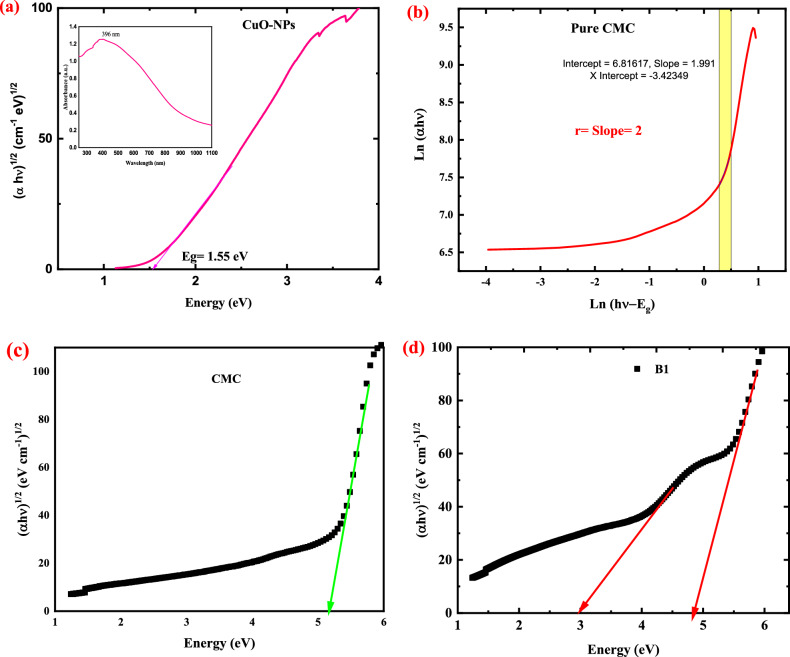

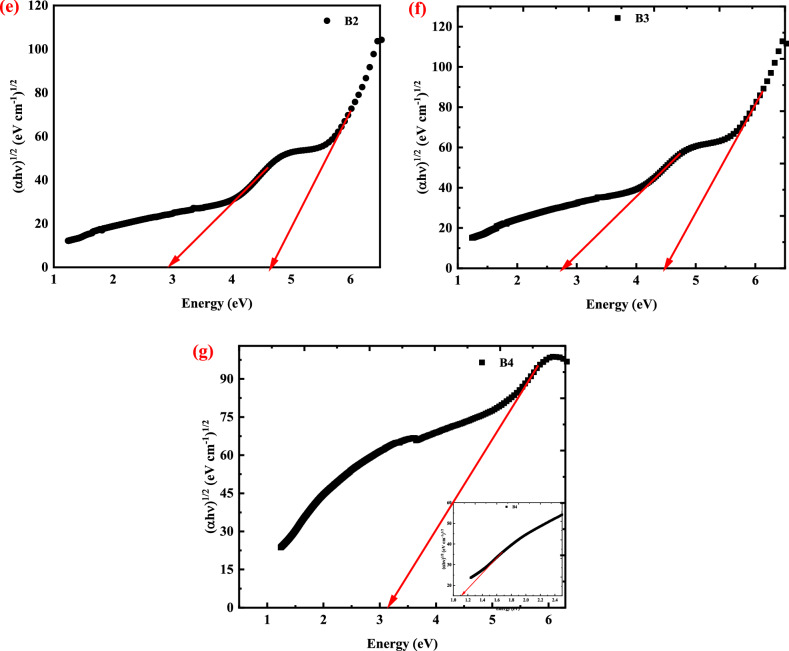
(**a**) variation of (αhν)^2^ of CuO- NPs versus photon energy together with the absorption spectra of CuO-NPs in the inset of the figure (**b**) plot of ln (αhυ) versus ln (hυ-E_g_) for pure CMC and (**c: g**) presents the plot of (αhν)^1/2^ versus hν for pure CMC and CMC doped with 2, 4, 6 and 8 wt. % CuO-NPs.

E_g_ is calculated by projecting the straight portion of the (αhν)^1/^^2^ versus (hν) plot to the energy axis at αhν = 0 and assuming indirect transitions between the valence band and the conduction band in CuO (Fig. 6a). CuO-NPs have an estimated Eg value of 1.55 eV, which is comparable with CuO-NPs values reported in the literature^49,50^. In addition, Chand et al.^51^ found E_g_ values for CuO-NPs ranging from 2.90 to 3.60 eV, depending on the pH of the solution. In the inset of Fig. 6a, the absorption spectra of CuO-NPs are displayed as a function of λ, and a band of absorption appears around 396 nm. The position of the absorption band in these findings is dependent on the particle size of the CuO-NPs that have been produced^50,52^.

The transition type and the optical band gap value of CMC can be determined by dividing Tauc’s equation by the product of the differentiation, as described by the following equation^53^:

10 $$\frac{d\left[ln\left(\alpha h\upsilon \right)\right]}{d[h\upsilon ]}=\frac{r}{(h\upsilon -Eg)}$$

Figure 6b shows the dependence of ln (αhυ) on ln (hυ-E_g_) for Pure CMC. The value of r can be determined by plotting ln(αhυ) against ln(hυ-E_g_). For pure CMC, from the slope of Fig. 6b, r is nearly equal to 2 in Eq. (10). This implies that the fundamental shoulder of absorption for pure CMC is formed by the indirect allowed transitions.

Figure 6c presents the plot of (αhν)^1/2^ as a function of photon energy (hν) for pure CMC, while Fig. 6d–g, present the plot of (αhν)^1/2^ versus hν for CMC doped with 2, 4, 6, and 8 wt.% CuO-NPs, respectively. The gap energy is calculated for pure CMC and the nanocomposite films and listed in Table 5. From the table, pure CMC has only one indirect band gap, which is equal to 5.27 eV as shown in Fig. 6c, which is in good agreement with the reported data^53^. The addition of different weight percentages of CuO-NPs (2, 4, 6, and 8 wt.%) to CMC reduces the HOMO/LUMO bandgap of CMC from 5.27 to 3.14 eV during the filling process of the CMC with CuO-NPs.

The displacement of the adsorption edge and the decrease in gap energy with increasing filler content confirms the presence of an interaction between the Nano-filler and the polymer’s functional groups. The addition of different weight percentages of CuO-NPs (2, 4, 6 and 8 wt. %) to CMC reduces the indirect bandgap of CMC (5.27 eV) until reach to 1.13 eV (for the sample denoted B4), as seen in the inset of Fig. 6g. On this concern, the addition of CuO-NPs creates localized states inside the bandgap, the transition between such states results in a bandgap value less than the HOMO/LUMO bandgap. The formation of localized states inside the bandgap as a result of doping has been previously mentioned in Euvrard et al. (2018) and El-Naggar et al. (2022)^54,55^.

Due to the creation of localized states within the valence band of CMC, it was discovered that all of the manufactured samples have two indirect band gaps. The highest values correspond to the HOMO/LUMO gap, and the second to the onset gap.

Furthermore, as previously indicated in the FTIR section, this decrease can be explained in terms of the creation of hydrogen bonds between CuO-NPs and the pure CMC. The decrease in E_g_ of CMC films with increasing CuO-NPs concentration could also be explained by the formation of localized states in the band gap of the host polymer matrix, which operate as trapping and recombination centers. The comparatively wide bandgap of the pure CMC was decreased to match new optical and optoelectronic applications by this method of CuO-NPs filling. Because of the momentum conservation law, indirect band gap semiconductor materials are good candidates for UV-blocking applications. In indirect semiconductors, holes and electrons have different moments. Thus, to recombinantly fulfill the momentum conservation law, they need to do something with this uncompensated momentum. Electron and hole-pair momentum can be zero in direct band gap semiconductors, allowing them to simply recombinate. CuO-NPs filling as an environmentally benign material allows for the innovative purpose of customizing the optical bandgap of CMC.

### Urbach energy determination

**Figure 7:**
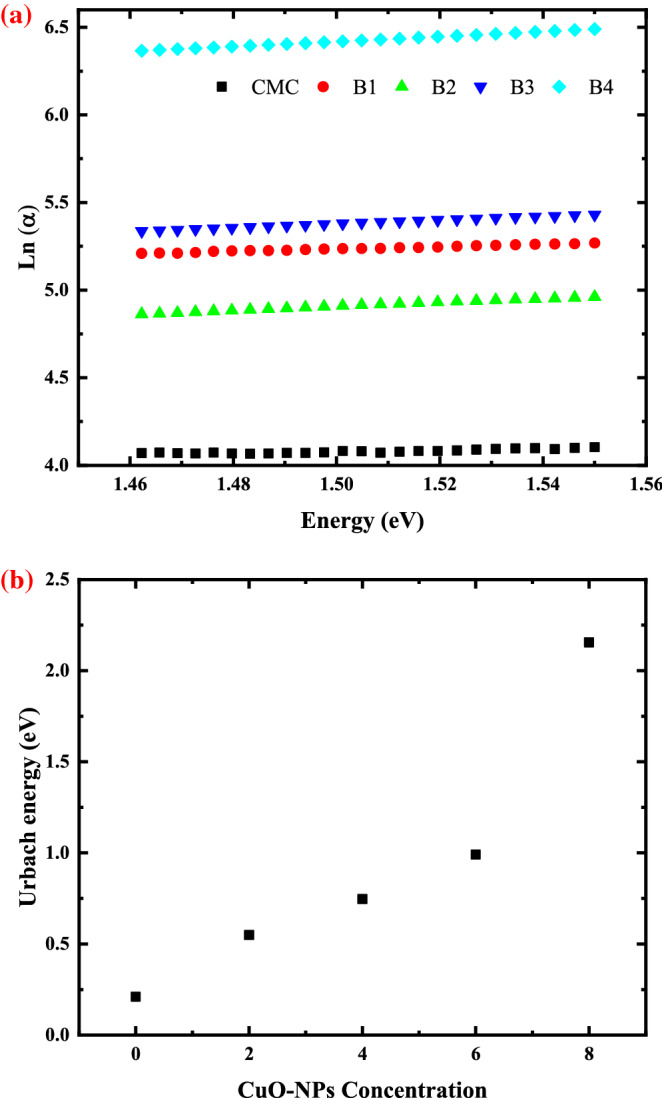
(**a**) Plotting of lnα as a function of photon energy (hυ) for pure CMC and CMC with different concentration of CuO-NPs and (**b**) variation of Urbach energy with CuO-NPs concentration.

The rise of non-crystalline ordering in the CMC film by CuO-NPs and the emergence of localized states in the prohibited band are supported by the absorption edge's reduction. This may be proven by using the following formula to calculate the Urbach energy (E_u_), which specifies the width of localized states within the forbidden gap^56^:

11 $$\mathrm{\alpha }={ \alpha_{o} }{e}^{(\frac{{E}_{U}}{h\upsilon })}$$

where α_o_ is a fixed number. Figure 7a presented the plot of lnα as a function of photon energy (hυ) for pure CMC and CMC with different concentrations of CuO-NPs, and Fig. 7b presented the variation of Urbach energy with CuO-NPs concentration. Figure 7a shows the linear connections for all as-fabricated films between ln(α) and photon energy (hυ). The Urbach energy increases with CuO-NPs concentration, as shown in Fig. 7b. The E_U_ of each sample was determined by fitting the plots linearly, and the results are shown in Table 6. The band tail widens from 0.21 eV (CMC) to 2.25 eV (sample B4). This finding supports the notion that when the CuO-NPs percentage in the CMC matrix increased, the degree of disorder rose and localized states in the forbidden band emerged, facilitating electron transitions^57^.

**Table 6:** Urbach energy of pure CMC and CMC with different concentrations of CuO-NPs (2, 4, 6 and 8wt.%).

Samples	Urbach energy (eV)
Pure CMC	0.21
B1	0.55
B2	0.75
B3	0.87
B4	2.25

### Evaluation of the refractive index and extinction coefficient

In optical communication and optical device design, the refractive index (n) is critical. The most common method for estimating the refractive index (n) is to employ reflectance (R) spectra, which have the following relationship with n^7^:

12 $$\mathrm{n}=((1+\mathrm{R})/(1-\mathrm{R}))+\sqrt{(4\mathrm{R}/{\left(1-\mathrm{R}\right)}^{2})-{\upkappa }^{2}}$$

**Figure 8:**
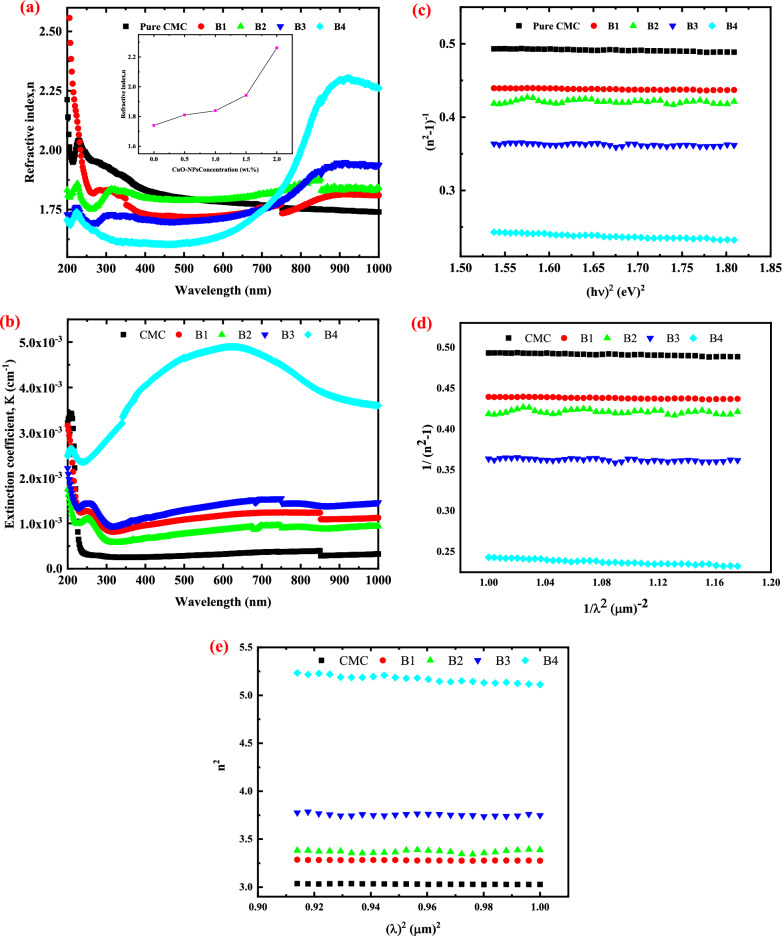
(**a**) Wavelength dependent refractive index (n) with the variation of n with CuO-NPs concentration (inset of the figure) (**b**) variation of extinction coefficient with the wavelength for CMC and CMC with different CuO-NPs concentration (**c**) the plot of 1/(n^2^-1) versus (hν)^2^ (**d**) 1/(n^2^-1) versus λ^-2^ and (**e**) n^2^ versus λ^2^ of pristine CMC and CuO-NPs filled CMC nanocomposite films.

Figure 8a depicts the relationship between n and wavelength (λ). The values of n dramatically decrease with increasing CuO-NPs content in most of the investigated range (200-750 nm), then increase with increasing CuO-NPs content in the nanocomposite films (as presented in the inset of Fig. 8a), where the intermolecular hydrogen bonding between CuO-NPs and the neighboring OH groups of CMC was thought to be responsible for this behavior, leading to an increase in the density of the films, and thus the films became more packed. The establishment of chemical bonds between the CMC chains and CuO-NPs and the emergence of certain localized states between the HOMO and LUMO bands of CMC, which result in a feasible smaller energy transition, can be linked to the reduction in the energy gap. Additionally, it may be connected to the rise in CMC's disorder level with rising CuO-NPs levels. Additionally, these findings agree well with the XRD analysis of the prepared films. Strong scientific interest exists in the relationships between the bandgap (E_Opt_) and refractive index (n). The material's transparency and response to electromagnetic waves were indicated by the refractive index. Due to its critical function in optical fiber technology, planer waveguides, and optoelectronics, its determination is crucial.

The extinction coefficient (k) is among the most important optical properties that affect the material absorption when radiation is absorbed. The extinction coefficient (k) was calculated using the following equation^7^:

13 $$\upkappa =\mathrm{\alpha \lambda }/4\uppi$$

As demonstrated in Fig. 8b, the extinction coefficient of CMC/CuO nanocomposite and pure CMC fluctuates with photon wavelength. As the concentration of CuO-NPs increased, the extinction coefficient of the CMC film increased. This behavior of the extinction coefficient can be explained by an increase in the number of charge carriers as a consequence of the rising absorption coefficient. In addition, the interaction between the energy carried in the samples and the incident light causes polarization of the medium charges, resulting in a loss of incident photon energy. This indicates that CuO-NPs will alter the structure of the CMC matrix.

### Optical dispersion parameters

The Wemple–DiDomenico single oscillator (WDD) model was also used to investigate the refractive index dispersion in the non-absorbing zone (hv < Eg). It is critical to investigate the dispersion performance of such polymeric nanocomposite for optics and similar applications^58^. Thus, the prepared pristine CMC and CuO-NPs filled CMC nanocomposite films' oscillator (E_o_) and dispersion (E_d_) energies were calculated. The oscillator energy (E_o_) represents the average bandgap of the material, whereas the dispersion energy (E_d_) refers to the average strength of the interband transition, which is given by

14 $${n}^{2}\left(h\upsilon \right)-1={E}_{o}{E}_{d}/\left({E}_{o}^{2}-{\left(h\upsilon \right)}^{2}\right)$$

where E_o_ and E_d_, stand for the single oscillator and dispersion energies, respectively. It is important to note that the WDD model is applicable in the region where the incident photons' energy (hv) is less than the optical bandgap and dependent on the refractive index (n) of these nanocomposite films. The single term "Sellmeier oscillator relations" could be used to derive a number of new optical constants at this area where the absorption vanishes (i.e., at an infinite wavelength)^58–60^:

15 $${n}^{2}={\varepsilon }_{l}-({e}^{2}N/4{\pi }^{2}{\varepsilon }_{o}{m}^{*}{c}^{2}) {\lambda }^{2}$$

16 $$\frac{{n_{\infty }^{2} - 1}}{{n^{2} - 1}} = 1 - \left( {\frac{{\lambda _{0} }}{\lambda }} \right)^{2}$$

17 $${\varepsilon }_{\infty }= {n}_{\infty }^{2}$$

where, ${{\varvec{\varepsilon}}}_{{\varvec{l}}}$ stands for the lattice dielectric constant, ${{\varvec{\varepsilon}}}_{\boldsymbol{\infty }}$ for the infinite dielectric constant, ***e*** for the electronic charge, ***C*** for the speed of light, ${{\varvec{\varepsilon}}}_{{\varvec{o}}}$ for the dielectric constant of free space, ***N*** for the concentration of free carriers, and m* for the effective mass.

E_o_ and E_d_ of the pure and filled CMC films were determined from the plots of 1/(n^2^-1) as a function of (hv)^2^, as shown in Fig. 8c, in accordance with Eq. (14). The slope is equal to -1/(E_o_ E_d_), while the intercepts with the vertical axis of the linear sections equal to (E_o_/E_d_). Table 7 contains the obtained E_o_ and E_d_ values. It should be noted that the CMC film's E_o_ value is 5.00 eV, which is higher than that of any filled nanocomposite film. Additionally, E_o_ falls to 2.86 eV as the nanofillers' content increases to 8 wt.%, while the E_d_ value increased when the concentration of the nanofillers increased to 8 wt.%. Since E_o_ is related to the optical energy bandgap and behaves similarly to it as explained above using Tauc's equation, these results are understandable. In other words, as filling introduces localized states and defects into the nanocomposite films as opposed to the pristine CMC, E_o_ drops as the concentration of nanofillers increases, just as E_g_ does. The rise in the degree of disordering and subsequent reinforcing of the optical transitions is what is responsible for the nanocomposite films' increased E_d_ values as compared to pristine ones^60^.

**Table 7:** Optical dispersion parameters of pristine CMC and CuO-NPs filled CMC films.

CMC loaded with CuO-NPs (wt.%)	E_O_ (eV)	E_d_ (eV)	${\varepsilon }_{\infty }$	${\varepsilon }_{l}$	N/M* (g^-1^ cm^-3^)	N (g/cm^3^)
0	5.00	9.32	2.93	3.16	4.41 × 10^55^	4.02 × 10^28^
2	5.51	11.89	3.16	3.42	4.85 × 10^55^	4.41 × 10^28^
4	4.53	9.88	3.19	3.49	4.37 × 10^55^	3.98 × 10^28^
6	4.57	11.59	3.57	3.90	4.89 × 10^55^	4.45 × 10^28^
8	2.86	9.60	4.26	6.34	3.88 × 10^56^	3.54 × 10^29^

The standard Sellmeier oscillator model (Eqs. 15, 16, and 17) is used to calculate additional optical parameters, such as the infinite dielectric constant (${{\varvec{\varepsilon}}}_{\boldsymbol{\infty }}$**),** lattice dielectric constant (${{\varvec{\varepsilon}}}_{{\varvec{l}}}$), the ratio of free carrier concentration to effective mass (N/m*), and the free carrier concentration (N) for the produced films in the non-absorption area in addition to the dispersion parameters determined above. According to Fig. 8d, the infinite dielectric constant was calculated by linearly fitting 1/(n^2^-1) plots against λ^-2^ for the unfilled and filled CMC films. The graph shows that the intercept of the fitted lines is related to ${{\varvec{\varepsilon}}}_{\boldsymbol{\infty }}$ by, ${{\varvec{\varepsilon}}}_{\boldsymbol{\infty }}$**=** $\frac{1}{\mathrm{intercept}}$  + 1^61^.

Additionally, as illustrated in Fig. 8e, plotting is used to determine the ${{\varvec{\varepsilon}}}_{{\varvec{l}}}$ values represented by the intercepts of the extensions of the fitted linear lines in these plots, whereas N/m* values were calculated from the slops of these fitted lines. Table 7 contains a list of all the obtained optical constants (${\varepsilon }_{\infty }$, ${\varepsilon }_{l}$, N/m*, and N). It is obvious that the optical properties of the manufactured nanocomposite films are directly influenced by the concentration of the nanofillers. Additionally, it was determined that the filling method significantly improves the infinite dielectric constant, lattice dielectric constant, the ratio of free carrier concentration to effective mass, and the free carrier concentration. For example, for 8 wt.% of CuO filled CMC nanocomposite films as compared to the pure one, the ratio of free carrier concentration to the effective mass (N/m*) increased by 11%, and the lattice dielectric constant increased by nearly 50%.

Additionally, it was noticed that the lattice dielectric constant for all prepared nanocomposite films is greater than the infinite one. This conduct is a result of the free carriers' contribution as presented in Table 7. Table 8 presents all of the optical parameters determined for pure CMC and CMC filled with 2, 4, 6, and 8 wt. % of CuO-NPs, including the onset band gap and HOMO/LUMO band gap using the Tauc and ASF models, respectively, the Urbach energy, T% at 300 and 600 nm, oscillation and dispersion energies, infinite and lattice dielectric constants, and the free carrier concentration (N). According to Table 8, adding different weight percentages of CuO-NPs to the CMC matrix results in a decrease in optical bandgap energy (both onset and HOMO/LUMO gaps), an increase in Urbach energy, a decrease in transmittance percent, a decrease in oscillation energy, an increase in dispersion energy, infinite and lattice dielectric constants, and free carrier concentration. Decreasing the T% to 1.10% and 2.06% at 300 and 600 nm, respectively, confirms that CMC /CuO nanocomposite films can be used as broadband UV–visible filters. Thus, CuO-NPs filled CMC nanocomposite films are now qualified as viable options for several optical and storage applications based on our findings, such as blocking material for UV radiation.

**Table 8:** Summarizes all of the optical parameters of CMC and CMC/ CuO nanocomposite films.

Absorption Parameters					Dispersion Parameters							
CuO wt.%	Tauc Model		ASF Model		Eu (eV)	T% at 300 nm	T% at 600 nm	E_O_ (eV)	E_d_ (eV)	${\varepsilon }_{\infty }$	${\varepsilon }_{l}$	N (g^1^cm^3^) × 10^28^
	Onset Gap (eV)	HOMO/LUMO gap (eV)	Onset Gap (eV)	HOMO/LUMO gap (eV)								
0	–	5.27	–	5.29		70.40	80.87	5.00	9.32	2.93	3.16	4.02
2	2.97	4.87	2.99	4.86	0.55	34.73	48.19	5.51	11.89	3.16	3.42	4.41
4	2.95	4.66	3.06	4.68	0.75	48.88	61.36	4.53	9.88	3.19	3.49	3.98
6	2.78	4.49	2.75	4.52	0.87	31.20	42.55	4.57	11.59	3.57	3.90	4.45
8	1.13	3.14	1.04	3.17	2.25	1.10	2.06	2.86	9.60	4.26	6.34	35.40

## Conclusion

By using the solution casting approach, CMC films loaded with different concentrations (0–8 wt.%) of CuO-NPs were created. The amorphous character of the pure CMC film and the formation of copper oxide NPs in the tenorite phase (CuO) are both visible in the XRD examination. The host matrix's amorphous feature has increased as a result of the loading with CuO-NPs. This increase is thought to be due to the increased disordering of CMC with CuO filling. FTIR results confirm the formation of hydrogen bonding between CMC and CuO-NPs, which reflects the formation of a nanocomposite. HRTEM images reveal that CuO nanopowder is formed as a result of (agglomerated) CuO aggregates, which are made up of clusters of considerably smaller CuO crystallites. The effect of CuO-NPs concentration on the optical properties of nanocomposite films has been investigated using UV–Vis-NIR spectra measurements. UV–Vis-NIR demonstrates that the optical transmittance of pure CMC film is greater than 80%, and it declines as CuO-NPs are added. The results showed that the addition of CuO-NPs reduced light transmission significantly in both the UV and visible light bands, thus CMC/ CuO nanocomposite films can be used a UV blocker. Due to the localized states produced, the loading of CuO-NPs reduces the optical bandgap of the host matrix. The addition of CuO-NPs improves the refractive index of the CMC. Using the WDD model, the dispersion parameters of the unfilled and filled CMC nanocomposite films have been examined. This outcome might be understood in terms of the increase in the dispersion and reflection processes compared to pristine CMC film. The dispersion energy, oscillation energy, infinity dielectric constant, lattice dielectric constant, and other parameters are all significantly impacted by the CuO-NPs loading. According to the data, CMC loaded CuO-NPs are recommended for use in various optical and storage applications.

## Data Availability

The data will be available on request, contact person Medhat A. Ibrahim (Email: medahmed6@yahoo.com).
